# Challenges encountered in employing a low tidal volume ventilation strategy in patients at risk of ARDS

**DOI:** 10.1186/2197-425X-3-S1-A270

**Published:** 2015-10-01

**Authors:** R Fisher, A Selman, Z Brummell, BO Rose

**Affiliations:** University Hospital Lewisham, Critical Care Unit, London, United Kingdom

## Introduction

Ventilator strategies that use tidal volumes (TVs) limited to 6 ml/kg IBW (ideal body weight) have been shown to reduce mortality in patients with ARDS (acute respiratory distress syndrome). [1] It is suggested that using these same low tidal volumes in other critically ill patients may reduce the progression to ARDS. [2, 3] An initial audit in the ICU at University Hospital Lewisham demonstrated that patients without ARDS were receiving mandatory ventilation in excess of 8 ml/kg IBW >40% of the time.

## Objectives

To assess the effectiveness of a new protocol and education program in reducing rates of excessive TV ventilation in a general ICU.

## Methods

Data was collected on all ventilated patients without ARDS during three 21-day periods: an initial audit (Febuary 2013); a re-audit following the initiation of the protocol and education program (June 2013); a subsequent re-audit following redrafting of the protocol and introduction of PRVC (Pressure Regulated Volume Control) as the default mode of mandatory ventilation (November 2013).

Mode of ventilation and measured TV were recorded hourly by the patient's bedside nurse. Investigators estimated IBW by using the patient's ulna length as a surrogate of height.

## Results

During the initial audit period 17 patients received a total of 1393 hours of mandatory ventilation, of which 40.4% of TVs were >8 ml/kg IBW. More than half of the patients studied received TVs >8 ml/kg IBW for the majority of the study period.

During the first re-audit period 16 patients received a total of 794 hours of mandatory ventilation, of which 40.4% was >8 ml/kg IBW (p = 1).

**Figure 1:**
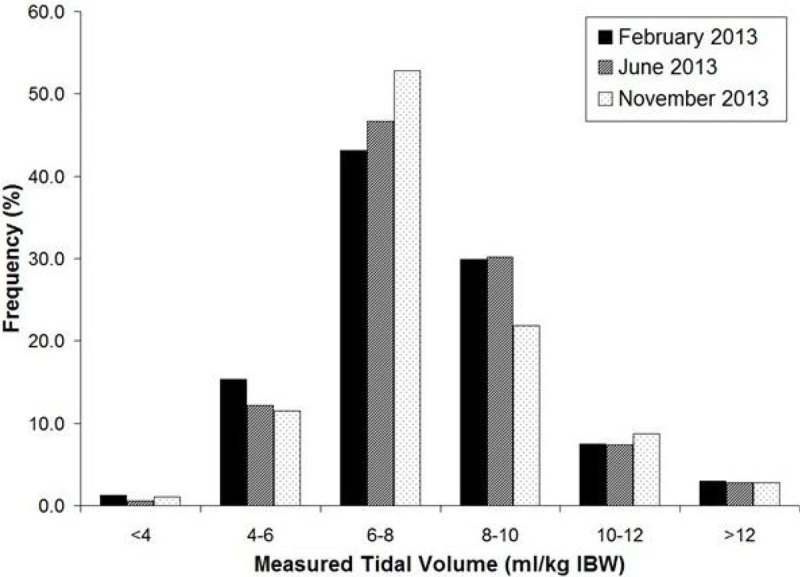
**Proportion of recorded TVs by ml/kg IBW.**

During the second re-audit period 15 patients received a total of 846 hours of mandatory ventilation, of which 33.4% was >8 ml/kg IBW (p = 0.001) (Figure 1).

## Conclusions

Introduction of a formalised protocol supported by an education program failed to decrease the rate of excessive TV ventilation in ICU patients without ARDS. The addition to the protocol of a default mode of ventilation that requires staff to set the target TV significantly reduced rates of excessive ventilation, however TVs >8 ml/kg IBW continued to be delivered a third of the time and there remains scope for improvement. These results highlight the difficulty in changing staff practice (both medical and nursing) in the ICU.
